# VEGF-A Stimulates STAT3 Activity via Nitrosylation of Myocardin to Regulate the Expression of Vascular Smooth Muscle Cell Differentiation Markers

**DOI:** 10.1038/s41598-017-02907-6

**Published:** 2017-06-01

**Authors:** Xing Hua Liao, Yuan Xiang, Hui Li, De Liang Zheng, Yao Xu, Cheng Xi Yu, Jia Peng Li, Xiao Yu Zhang, Wei Bin Xing, Dong Sun Cao, Le Yuan Bao, Tong Cun Zhang

**Affiliations:** 1 Institute of Biology and Medicine, Wuhan University of Science and Technology, Wuhan, 430000 P. R. China; 2 Key Laboratory of Industrial Fermentation Microbiology, Ministry of Education and Tianjin, College of Biotechnology, Tianjin University of Science and Technology, Tianjin, 300457 P. R. China

## Abstract

Vascular endothelial growth factor A (VEGF-A) is a pivotal player in angiogenesis. It is capable of influencing such cellular processes as tubulogenesis and vascular smooth muscle cell (VSMC) proliferation, yet very little is known about the actual signaling events that mediate VEGF-A induced VSMC phenotypic switch. In this report, we describe the identification of an intricate VEGF-A-induced signaling cascade that involves VEGFR2, STAT3, and Myocardin. We demonstrate that VEGF-A promotes VSMC proliferation via VEGFR2/STAT3-mediated upregulating the proliferation of markers like Cyclin D1 and PCNA. Specifically, VEGF-A leads to nitrosylation of Myocardin, weakens its effect on promoting the expression of contractile markers and is unable to inhibit the activation of STAT3. These observations reinforce the importance of nitric oxide and *S*-nitrosylation in angiogenesis and provide a mechanistic pathway for VEGF-A-induced VSMC phenotypic switch. In addition, Myocardin, GSNOR and GSNO can create a negative feedback loop to regulate the VSMC phenotypic switch. Thus, the discovery of this interactive network of signaling pathways provides novel and unexpected therapeutic targets for angiogenesis-dependent diseases.

## Introduction

As one of the major components of the aortic wall, vascular smooth muscle cell (VSMC) plays an important role in vessel remodeling during pregnancy, exercise, and vascular injury^1^. VSMCs express a unique repertoire of contractile markers specific to smooth muscle, such as smooth muscle alpha actin (αSMA), SM22, ACTA2, MHC, calponin and alpha-tropomysin^2^. When pathological conditions are present, VSMCs increase their proliferation, migration, and lose the “contractile” phenotypic, such as vasculopathy and atherosclerosis^3^. This phenotypic switch between the contractile and synthetic VSMC phenotypes is tightly controlled through a synergistic and coordinated molecular regulatory network^4, 5^. VEGF-A is a key regulator of vascular development, which is refering to not only the physiological process but also some pathological changes^6^. VEGF-A and its receptor (VEGFR) are known to be the major regulators of angiogenesis, in which the VEGF-A can modulate several cellular functions of endothelial cells including survival, proliferation, migration, and tube formation by binding to VEGFR2^7^. Activated VEGFR2 transduces the signal into endothelial cells through complex signaling cascades that mediate various cellular processes required for angiogenesis^8, 9^. Hypoxia and endothelin-1 induce VEGF expression in human vascular smooth muscle cells^10^. The increased VEGF mRNA was preceded by the enhancement of both TGF-β1 expression and reactive oxygen species (ROS) generation in the smooth muscle cell (SMC)^11^. VEGF may inhibit SMC proliferation via a mechanism that involves VEGF-induced NO production from the endothelium^12^. VEGF indirectly stimulates SMC proliferation and migration through stimulation of the expression of FGF-2 and VEGF, ultimately inhibits the expression of TGF-beta1 released by enhance endothelial cell (EC)^13^. The effects of VEGFRs on SMCs suggested that VEGF may play an important role in modulating the response of SMCs^14^.

VEGFA-induced VEGFR2 expression can enhance EC proliferation by exacerbating STAT3 activation, leading to pathological angiogenesis^15^. STAT3, play an important role in angiogenesis under both physiological and pathological conditions in addition to cell survival, proliferation, differentiation, and oncogenesis^16^. It also is essential for VEGF-A-induced lymphatic endothelial cell migration and tube formation^17^. VEGF stimulation induced STAT3 phosphorylation by a VEGFR2- and Src-dependent mechanism in cultured EC cells^18^. Further evidence indicated that STAT3, a key regulator of angiogenesis, is induced by proangiogenic factors such as HIF-1α and VEGF-A^10, 19^. In addition, VEGF-A-induced EC migration is mediated in part through activation of the JAK-STAT3 signal transduction pathway^20^. Hypoxia-induced activation of STAT3 transactivated the VEGF promoter and increased the expression of VEGF transcripts^19^. An activated STAT3 mutant (STAT3C) stimulates tumor angiogenesis by up-regulating VEGF expression, however, STAT3C-induced VEGF up-regulation is abrogated when the STAT3-binding site in the VEGF promoter is mutated^21^.

Phosphorylated STAT3 (pSTAT3) pSTAT3, a molecular hub for signal transduction pathways in EC, is able to regulate the activity of VEGF promoter and alter VEGF transcription^18^. STAT3 and myocardin can regulate the expression of VEGF and VEGF-STAT3-VEGF forms a positive feedback regulation to mudulate smooth muscle phenotype conversion^22^. Although the critical roles of VEGF-A/STAT3 in SMC cell proliferation are well recognized, the specific role of VEGF-A-VEGFR2-STAT3-induced VSMC cell phenotypic switch is still poorly understood and the mechanism by which Myocardin regulates VEGF-A-VEGFR2-STAT3-induced VSMC cell phenotypic switch is not known.

Herein, in this study, we characterize the participation of VEGFR2 in the regulation of VEGF-A-mediated VSMC cell proliferation. We demonstrate that activated STAT3, which has previously been predominantly linked to VSMC cell proliferation^22^, is expressed in VSMC cells and is a key mediator of VEGF-A-induced VSMC cell proliferation. Myocardin, a STAT3 interaction protein^22^, is a critical link in the VEGF-A-VEGFR2-STAT3 signaling pathway, undergoing nitrosylation and the effect on contractile markers expression is inhibited after treating with VEGF-A. The regulation of STAT3 by VEGF-A-VEGFR2 provides a link between Myocardin and STAT3 to regulate VSMC cell phenotypic switch. Interestingly, Myocardin positively modulates GSNOR expression, GSNOR can downregulate GSNO and meanwhile GSNO inhibits the effect on promoting the contractile markers expression by Myocardin via *S*-nitrosylation, thus creating a negative feedback loop to regulate the VSMC phenotypic switch.

## Results

### VEGFR2 Is Activated by VEGF-A and Is Required for VSMC Phenotypic Switch

VEGFR2 is known to mediate the full spectrum of VEGF responses in SMC cells^9^. However, the exact roles of VEGFR2 in regulating the VEGF-mediated VSMC phenotypic switch in T/G HA-VSMC cells are not known. To examine this, we firstly asked the following questions: Does the addition of VEGF to T/G HA-VSMC cell cultures result in VEGFR activation? And, if so, does VEGFR2 play a role in the VEGF-induced VSMC phenotypic switch?

The activation of VEGFR induced by VEGF-A was examined in T/G HA-VSMC by monitoring phosphorylation of the Y-1214 activation site in VEGFR2. Our results demonstrated that VEGFR2 was phosphorylated in a time- and dose-dependent manner. Phosphorylation of Y-1214 peaked at 48 hours after VEGF-A stimulation (Fig. S1A) and increased in a dose-dependent fashion over the VEGF-A concentration range of 25–100 ng/mL (Fig. S1B), which approximates physiologically relevant VEGF-A concentrations. The involvement of VEGFR2 in VEGF-A-induced T/G HA-VSMC cell proliferation was confirmed by treatment of cells with siRNAs of VEGFR2. T/G HA-VSMC cells were transfected with 1 of 3 different VEGFR2-specific siRNAs, or mixture of all three, and 72 hours later cell lysates were examined by Western blot analysis for levels of VEGFR2 protein. Each separate siRNA entity and the mixture of all three were found to be similarly effective at suppressing expression of endogenous VEGFR2 in T/G HA-VSMC cells (Fig. S1C). Likewise, transfection of cells with VEGFR2-specific siRNA resulted in a significant decrease of Cyclin D1 and PCNA expression in response to VEGF treatment (Fig. S1D). Besides, VEGF-A can enhance the expression of stem cell markers CD44 and Sox17 in VSMC (Fig. S2). Human VSMC may be stem cell-derived during the culture process. Therefore, inhibiting VEGFR2 activity by either siRNA or a specific inhibitor is significantly detrimental to VEGF-A-induced VSMC proliferation, indicating that VEGFR2 is activated downstream of VEGF-A in T/G HA-VSMC cells and this activation is required for VEGF-A-stimulated VSMC phenotypic switch.

### STAT3, but Not STAT1 or STAT5, Is Activated by VEGF-A

**Figure 1:**
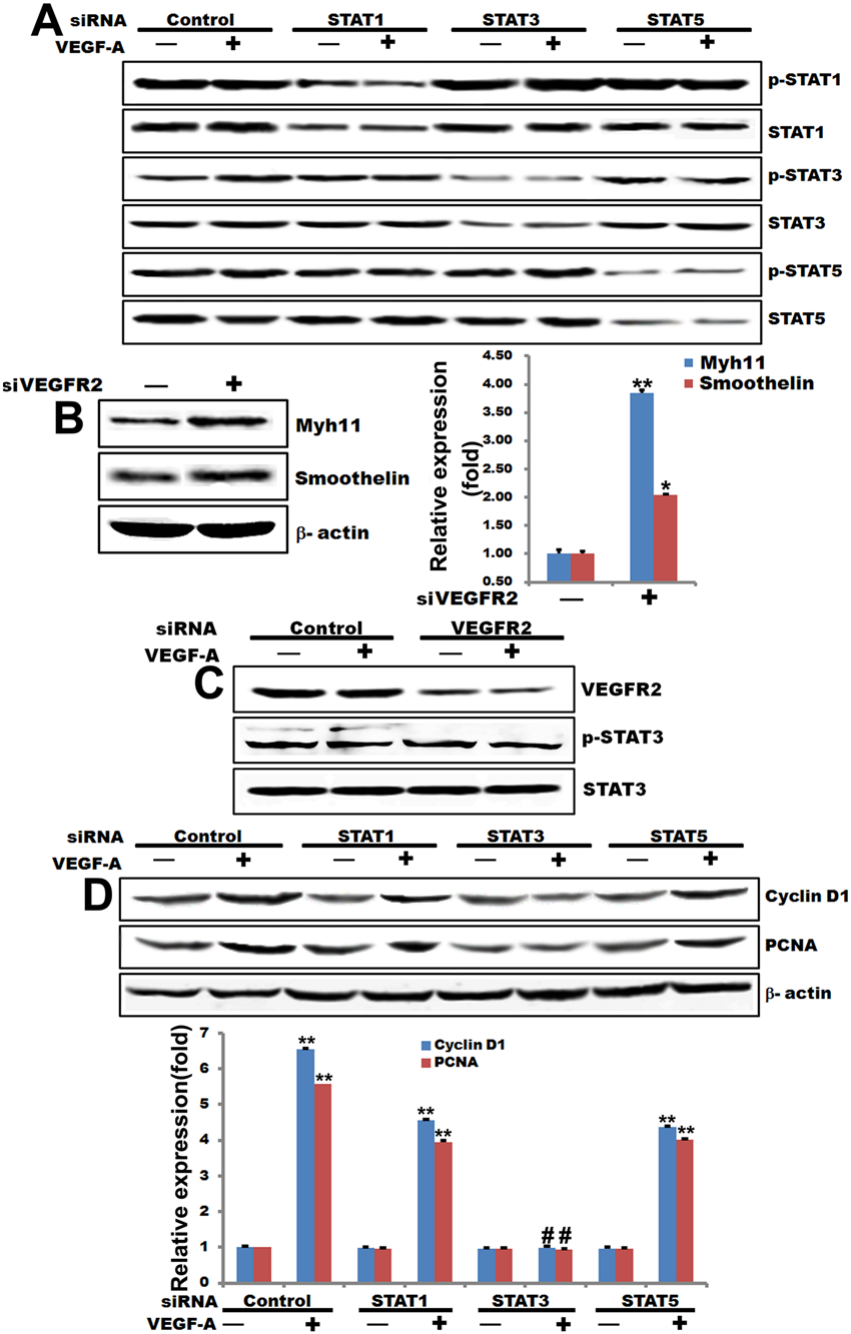
STAT3 acts as a downstream mediator for VEGFR2 activation induced by VEGF-A. (**A**) Western blot analysis of T/G HA-VSMC cells transfected with siRNAs for STAT1, STAT3, or STAT5 and treated with 100 ng/mL VEGF-A for 48 hours. (**B**) Western blot analysis of T/G HA-VSMC cells transfected with siRNAs for Myh11 and Smoothlin and quantified the western blot data by Quantity One software. β-actin is a loading control. **p < 0.01, *p < 0.05. n = 3. (**C**) Western blot analysis of transfected with a mixture of VEGFR2 siRNA and treated with100 ng/mL VEGF-A for 48 hours (**D**) Western blot analysis of Cyclin D1 and PCNA protein level in T/G HA-VSMC cells with siRNAs for STAT1, STAT3, or STAT5 and treated with 100 ng/mL VEGF-A for 48 hours and quantified the western blot data by Quantity One software. β-actin is a loading control. **p < 0.01, *p < 0.05, ^#^ *p* > 0.05. n = 3.

In human, the STAT family is consisting of seven members, STAT 1–4, STAT5A, STAT5B and STAT6. In general, STAT3 and STAT5A/B promote oncogenesis, while STAT1 has opposing effects^23^. However, the exact role of STATs in VSMC proliferation remains unclear. To determine which STAT is involved in VEGF-A-induced VSMC proliferation, we screened the activities and expression levels of STAT1, STAT3, and STAT5 after treatment of T/G HA-VSMC cells with VEGF-A. Our previous research showed that VEGF-A (100 ng/mL) potently induced STAT3 activities in T/G HA-VSMC cells and presented in a dose-dependent manner^22^. When comparing the time course of STAT3 activation to that of VEGFR2, the peak lagged behind VEGFR2 (Fig. S1A), suggesting that STAT3 may act downstream of VEGFR2 to promote the proliferation events induced by VEGF-A in T/G HA-VSMC cells. To determine which STAT protein was activated by VEGF-A in these cells, we detected the phosphorylation of STATs by Western blot analysis. Interestingly, only phosphorylation of STAT3 (but not STAT1 or STAT5) was increased after treatment of T/G HA-VSMC cells with VEGF-A (Fig. 1A). To confirm the finding that STAT3 is activated by VEGF-A, we transfected cells with siRNAs designed for each of the STAT1, STAT3 and STAT5 sequences and then tested the effect on suppressing VEGF-A-induced STAT1, STAT3 and STAT5 activation. VEGF-A significantly enhanced STAT3 phosphorylation in the cells transfected with control siRNAs but has no effects on the STAT1 and STAT5 phosphorylation in the cells transfected with STAT1 and STAT5 siRNAs, respectively. However, the increase of STAT3 phosphorylation was abrogated in the cells transfected with STAT3 siRNA (Fig. 1A). These results suggest that different STAT isoforms might have different cellular functions in VSMC cells because they are being differentially regulated under the same conditions, with STAT3 specifically mediating effects downstream of the VEGF-A signaling.

### STAT3 Acts as a Downstream Mediator of VEGF-A -Induced VSMC Phenotypic Switch

**Figure 2:**
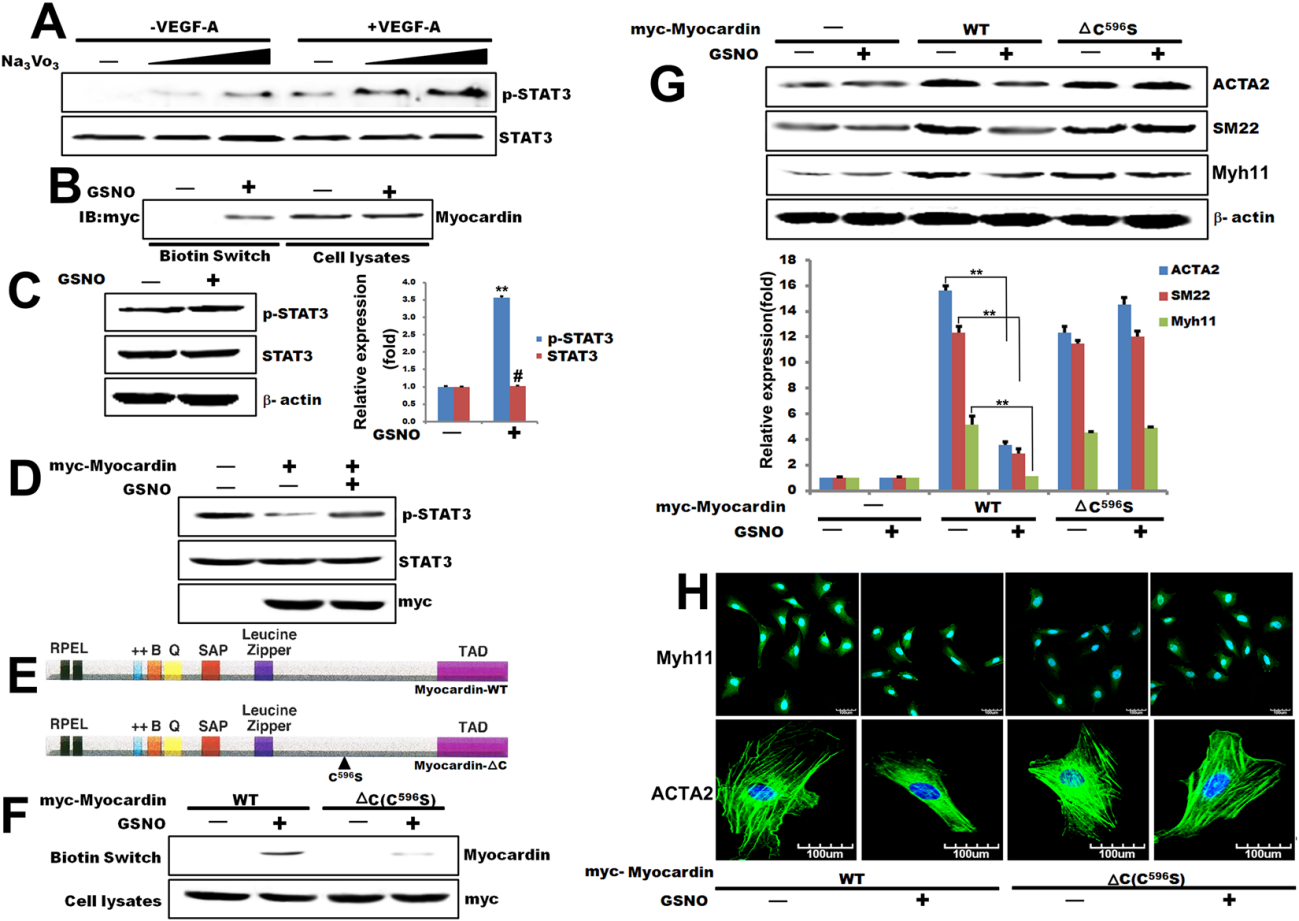
Myocardin is nitrosylated by nitric oxide. (**A**) Western blot analysis to detect phosphorylated STAT3 and total STAT3 protein in T/G HA-VSMC cells pretreated with Na3VO4 (10, 100, and 500 μM) and then treated with 100 ng/mL VEGF-A for 48 hours. (**B**) Results of a biotin switch assay on HEK293T cells transfected with Myc-Myocardin and treated with the nitric oxide donor nitrosoglutathione (GSNO) (500 μM) for 10 min at room temperature. (**C**) Western blot analysis to detect phosphorylated STAT3 and total STAT3 protein in T/G HA-VSMC cells pretreated with GSNO (500 μM) for 10 min at room temperature and quantified the western blot data by Quantity One software. β-actin is a loading control. **p < 0.01, ^#^ *p* > 0.05. n = 3. (**D**) Results of an *in vitro* phosphatase assay using active STAT3 protein as the substrate on lysates of HEK293T cells transfected with Myc-Myocardin. (**E**) Schematic structure of Myocardin mutant constructs. (**F**) Biotin switch analysis of HEK293T cells transfected with the indicated Myocardin-WT or Myocardin-ΔC and then treated with 50 μM GSNO for 10 min at room temperature. (**G**) Western blot analysis of ACTA2, SM22 and Myh11protein level in T/G HA-VSMC cells transfected with Myocardin-WT or Myocardin-ΔC then treated with 200 μM GSNO for 10 min and quantified the western blot data by Quantity One software. β-actin is a loading control. **p < 0.01. n = 3. (**H**) Immunofluorescence technique of Myh11and ACTA2 protein level in T/G HA-VSMC cells transfected with Myocardin-WT or Myocardin-ΔC then treated with 200 μM GSNO for 10 min.

VEGF-A promotes endothelial cells proliferation and angiogenesis^7^. Our data indicated that VEGFR2 activation is necessary for phenotypic switch of VSMC (Fig. S1), yet the mechanisms whereby VEGFR2-dependent regulation of VEGF signaling in VSMC phenotypic switch is unclear. To further confirm STAT3 is the target for VSMC cell proliferation by VEGF-A-induced VEGFR2 activation, VEGFR2 siRNAs were transfected into T/G HA- VSMC cells to knockdown endogenous VEGFR2 expression. As shown our results (Fig. 1B), knocked out VEGFR2 enhanced the markers (SM-MHC(Myh11) and smoothelin) of the differentiation of VSMCs. STAT3 was activated by VEGF-A in the cells transfected with control siRNA, but it was inhibited in the cells transfected with VEGFR2 siRNA (Fig. 2C). These results support the prediction that VEGFR2 is a required mediator for STAT3 activation induced by VEGF-A. STAT3 is activated upon VEGF stimulation of EC *in vitro* and *in vivo* by a VEGFR2-dependent or Src-dependent mechanism. In addition, the STAT3 activation can mediate Bcl-2 induction by VEGF-A^18^. However, the role of STAT3 in VSMC Phenotypic Switch has not yet been evaluated. To test the importance of STAT3 in complex cell processes, we examined the effect of STATs on the expression of the proliferation genes of VSMC. T/G HA- VSMC cells were transfected with STAT1 siRNA, STAT3 siRNA, STAT5 siRNA, or control siRNA. After 72 hours, cells were treated in medium containing 100 ng/mL VEGF-A. As shown in Fig. 1D, VEGF-A significantly enhanced the expression of proliferative marker Cyclin D1 and PCNA in the cells transfected with control, STAT1, and STAT5 siRNAs. However, the expression of Cyclin D1 and PCNA was abrogated in the cells transfected with STAT3 siRNA. These data once again implicate that STAT3 is the critical STATs moiety involved in VEGF-A-dependent VSMC phenotypic switch.

### Myocardin Is Inhibited by S-Nitrosylation to Affect the Differentiation of VSMC

The preceding experiments indicated that VEGFR2 and the downstream effector STAT3 are required for VEGF-A-induced VSMC cell proliferation. However, the molecular mechanisms of VEGF-A-VEGFR2-STAT3 regulation VSMC phenotypic switch are not intuitively obvious.

To explore the potentially regulatory mechanisms, we considered the possibility that regulation of STAT3 by VEGFR2 was determined by suppression of dephosphorylation activities. When cells were incubated with sodium orthovanadate, an inhibitor of protein tyrosine and dual-specificity protein phosphatases, STAT3 phosphorylation increased both under basal conditions and, most prominently, after VEGF-A treatment (Fig. 2A). These observations suggested that STAT3 is phosphorylated by VEGF-A.

Post-translational modification of proteins by S-nitrosylation serves a major model in mammalian cells and a growing body of evidence has shown that transcription factors and referring activating pathways are primary targets^24^. Myocardin is regarded as key mediators of smooth muscle phenotypic^25^. Our previous studies have demonstrated that STAT3 regulates VSMC phenotypic switch by interaction with Myocardin^22^. An acid/base primary sequence motif was first proposed by Stamler *et al*., who suggested that the cysteines which are targets for Snitrosylation are often flanked by acidic or basic groups [(K, R, H, D, E) C(D, E)]^26^. We found that Cys^596^ could be nitrosylated with an acid/base motif in its primary sequence by bioinformatics software. Located within the N-terminal of Myocardin, the Cys^596^ residue is highly sensitive to oxidation because of its low pKa, and this oxidation may be required for the decreased Myocardin activity. Given that VSMC itself has the ability to generate NO and in some circumstances can regulate protein function via *S*-nitrosylation of cysteine residues, we tested (*1*) whether Myocardin could be *S*-nitrosylated by nitric oxide and (*2*) whether *S*-nitrosylation of Myocardin would affect its ability to regulate STAT3 activity. An *in vitro* biotin switch nitrosylation assay demonstrated that Myocardin could indeed be *S*-nitrosylated by the exogenous nitric oxide donor nitrosoglutathione (GSNO) (Fig. 2B). As shown our results (Fig. 2C), GSNO did not affect the expression of STAT3 and the phosphorylation of STAT3. To determine whether this *S*-nitrosylation event affected STAT3 activity via Myocardin *S*-nitrosylation, we tested the phosphorylation level of STAT3 in Myocardin-overexpressed cells. The ectopically expressed Myocardin decreased the phosphorylation of STAT3 (Fig. 2D). However, nitrosylation of Myocardin by GSNO weaken the inhibition of the phosphorylation of STAT3 (Fig. 2D). To determine whether Cys^596^ was the targeted residue of Myocardin for nitrosylation, mutant constructs of Myocardin were generated (Fig. 2E). As shown in Fig. 2F, Myocardin-WT was strongly nitrosylated by GSNO. However, the nitrosylation of Myocardin-ΔC(C^596^S) by GSNO was markedly attenuated comparing to that of Myocardin-WT (Fig. 2F). Expression of Myocardin-WT or Myocardin-ΔC (C^596^S) in T/G HA-VSMC cells significantly promoted the expression of ACTA2, SM22 and Myh11; treating the overexpression Myocardin-WT T/G HA-VSMC cells with GSNO significantly decreased the expression of the contractile markers ACTA2, SM22 and Myh11; however, treating the overexpression Myocardin-ΔC (C^596^S) with GSNO the expression was not inhibited (Fig. 2G). As shown in Fig. 2H, treating GSNO in overexpression Myocardin-WT in T/G HA-VSMC cells significantly decreased the expression of Myh11 and ACTA2; however, treating GSNO in overexpression Myocardin-ΔC (C^596^S) in T/G HA-VSMC cells was not inhibited. Collectively, these observations indicated that the Cys596 locus in the catalytic domain of Myocardin is nitrosylated and Myocardin protein with S-nitrosylation decreases the effect on promoting the expression of ACTA2, SM22 and Myh11, providing mechanistic support that Myocardin is the mediator for regulation of STAT3 activity by nitric oxide during VSMC phenotypic switch.

### Myocardin S-Nitrosylation Induced by VEGF-A Is Required for STAT3 Activation in VSMC Phenotypic Switch

**Figure 3:**
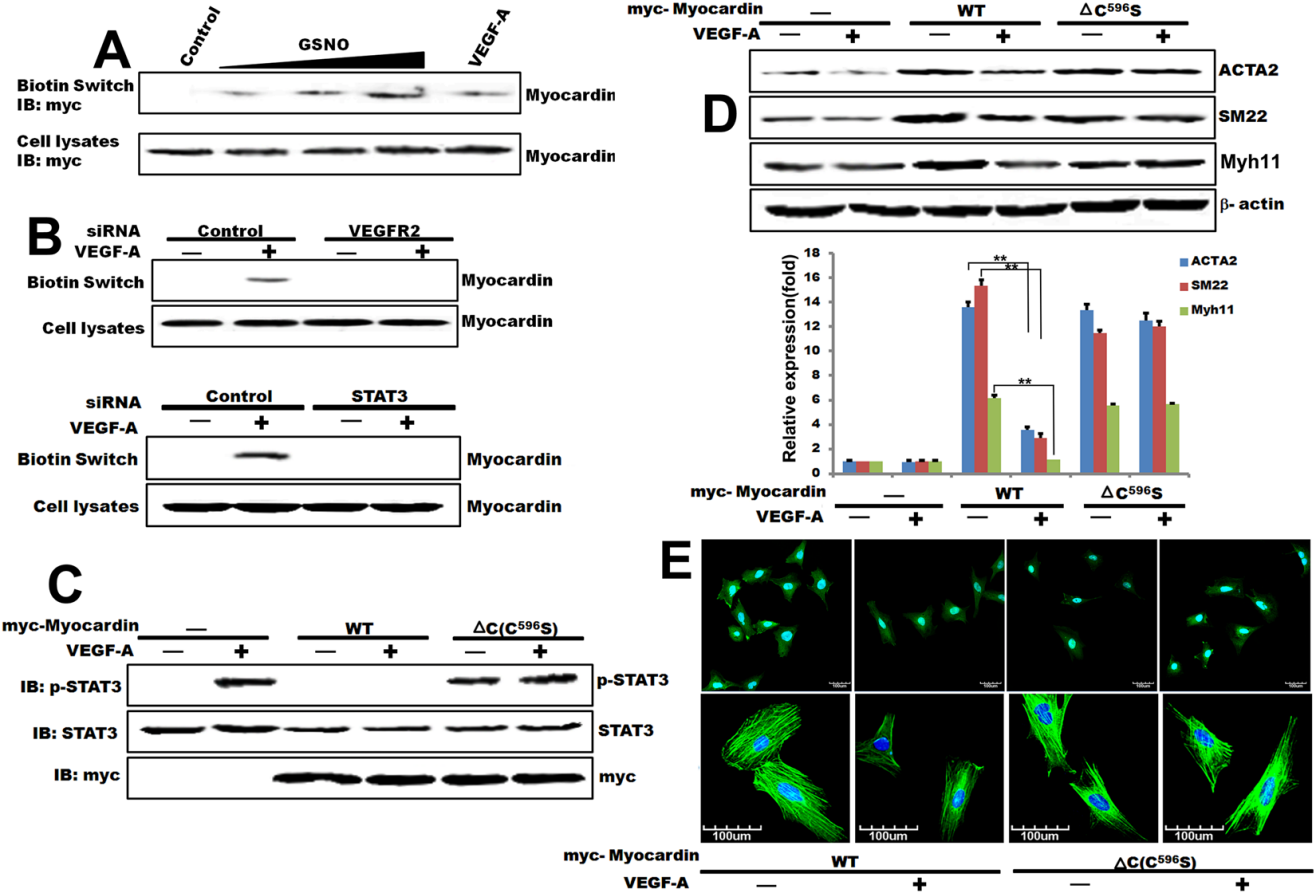
Myocardin nitrosylated at Cys596 is required for STAT3 activation in VSMC phenotypic switch by VEGF-A. (**A**) Biotin switch analysis of T/G HA-VSMC cells transfected with Myc-tagged Myocardin and followed by treatment with 200 μM GSNO for 10 min or 100 ng/mL VEGF-A for 48 hours (**B**) Biotin switch analysis of T/G HA-VSMC cells transfected with a 100 pmol of VEGFR2 or STAT3 siRNA mixture and 2 μg of Myc-tagged Myocardin and subsequently treated with 100 ng/mL VEGF-A for 48 hours. (**C**) Western blot analysis of T/G HA-VSMC cells transfected with Myocardin-WT or Myocardin-ΔC and activated with 100 ng/mL VEGF-A for 48 hours. The phosphorylated STAT3 and total STAT3 protein was determined by Western blot analysis in the cell lysates. (**D**) Western blot analysis of ACTA2, SM22 and Myh11 protein level in T/G HA-VSMC cells transfected with Myocardin-WT or Myocardin-ΔC then treated with 100 ng/mL VEGF-A for 48 hours and quantified the western blot data by Quantity One software. β-actin is a loading control. **p < 0.01. n = 3. (**E**) Immunofluorescence technique of Myh11 and ACTA2 protein level in T/G HA-VSMC cells transfected with Myocardin-WT or Myocardin-ΔC then treated with 100 ng/mL VEGF-A for 48 hours.

Because our *in vitro* data suggested that Myocardin is nitrosylated by nitric oxide, and this nitrosylation inhibits its activity, we next tested the relationship between VEGF-A stimulation and Myocardin *S*-nitrosylation in T/G HA- VSMC cells. For a first step, we investigated whether the treatment of VEGF-A in T/G HA-VSMC cells would induce *S*-nitrosylation of Myocardin. The result showed that VEGF-A can increase Myocardin nitrosylation (Fig. 3A), similar to the increase of that observed previously with GSNO. Additionally, VEGFR2 siRNA was used to investigate the effect on Myocardin nitrosylation when treating T/G HA-VSMC cells with VEGF-A. T/G HA-VSMC cells were transfected with VEGFR2 siRNA, control siRNA and Myc-tagged Myocardin. After 72 hours, the cells were treated with VEGF-A, and we performed a biotin switch assay. Knockdown of VEGFR2 blocked Myocardin *S*-nitrosylation after VEGF-A treatment, comparing to control siRNA (Fig. 3B). Next, we used STAT3 phosphorylation as a read out for determining whether VEGF-A activation by STAT3 could be inhibited by Myocardin. We determined that the VEGF-A-induced STAT3 activation was substantially inhibited by wildtype of Myocardin, but not by Myocardin mutant type-ΔC(C^596^S) (Fig. 3C). Interestingly, when cells that expressed Myocardin-ΔC(C^596^S) block the endogenous Myocardin level, STAT3 activity increased even under unstimulated conditions (Fig. 3C). Similarly, the full-length of Myocardin failed to decrease STAT3 activity; instead, STAT3 activity was increased by treatment with VEGF-A, compared with that without VEGF-A treatment (Fig. 3C). These results clearly demonstrated that, after VEGF-A treatment of T/G HA-VSMC cells, Myocardin is nitrosylated at Cys^596^ locus and this event in turn is required for VEGF-A-induced STAT3 activation. To confirm that Cys^596^ of Myocardin is the position of VEGF-A-induced nitrosylation, western blotting was used to detect the expression of VSMCs contractile markers ACTA2, SM22 and Myh11 in overexpressed Myocardin-WT or Myocardin-ΔC (C^596^S) cells that treating with VEGF-A. VEGF-A treatment inhibited the expression of ACTA2, SM22 and Myh11. Expression of Myocardin-WT or Myocardin-ΔC (C^596^S) in T/G HA-VSMC cells significantly promoted the expression of ACTA2, SM22 and Myh11, respectively; treating VEGF-A in overexpression Myocardin-WT in T/G HA-VSMC cells significantly decreased the expression of ACTA2, SM22 and Myh11; however, the VEGF-A in overexpression Myocardin-ΔC (C^596^S) in T/G HA-VSMC cells was not inhibited (Fig. 3D). This result strongly implied that endogenous Myocardin is the mediator for VEGF-A-dependent STAT3 activation. Myocardin *S*-nitrosylation inhibited the effect on promoting the expression of ACTA2, SM22 and Myh11 to regulate the VSMC differentiation. Together with the data presented previously that overexpressed Myocardin could be nitrosylated by nitric oxide, while VEGFR2 siRNA inhibited its nitrosylation, it supported that endogenous Myocardin could be nitrosylated by nitric oxide generated by VEGF-A, which in turn regulates VSMC phenotypic switch by altering the STAT3 activity.

To ascertain the functional consequences of Myocardin *S-*nitrosylation, we showed the morphological changes of the VSMC that transfected with the various Myocardin mutants by Immunofluoresence Staining. As shown in Fig. 3E, treating with VEGF-A in overexpression Myocardin-WT in T/G HA-VSMC cells significantly decreased the expression of ACTA2 and Myh11; however, the VEGF-A in overexpression Myocardin-ΔC (C^596^S) in T/G HA-VSMC cells was not inhibited. Collectively, these data indicated that Myocardin nitrosylation by nitric oxide after VEGF-A induced VEGFR2 activation provides a critical checkpoint for STAT3 activation and subsequent VSMC proliferation. Furthermore, these studies articulate a mechanism that Myocardin is a key participant in the coordination of events mediated by STAT3 after VEGF-A activation in VSMC phenotypic switch.

### Myocardin, GSNOR and GSNO Created a Negative Feedback Loop to Regulate the VSMC Phenotypic Switch

Alcohol dehydrogenase class III (ADH III is also named S-nitrosoglutathione reductase (GSNOR)) blocks NO function by transformed S-nitrosoglutathione (GSNO) to NH3^27^. GSNOR, highly conserved from bacteria to humans, is extensively expressed in almost all organisms^28^. Given GSNOR reduced GSNO to NH3 and Myocardin *S*-nitrosylation inhibited the expression of ACTA2 and SM22 to regulate the of VSMC cell differentiation, we want to investigate whether Myocardin affects the level of GSNOR in T/G HA-VSMC cells.

**Figure 4:**
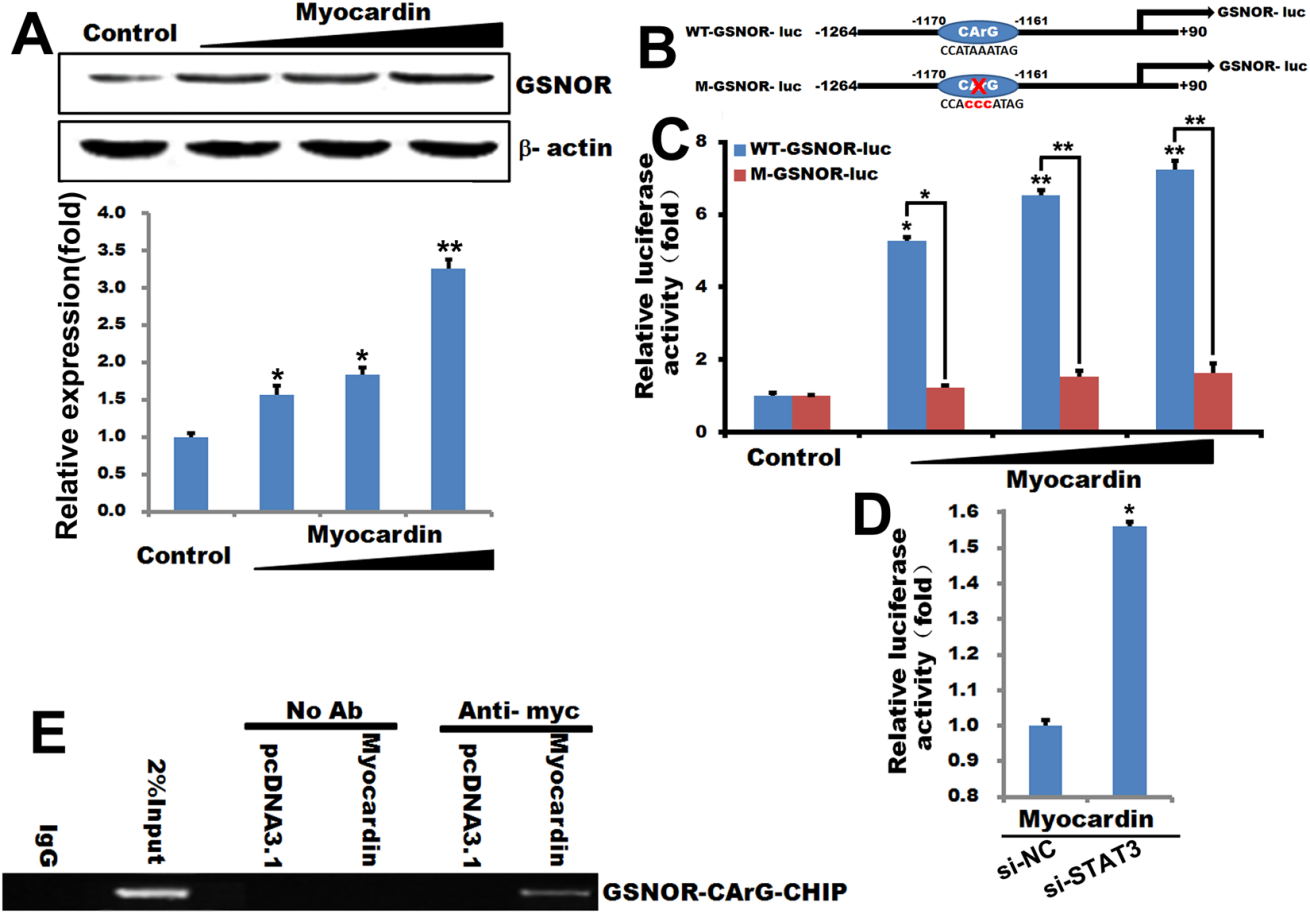
Myocardin-mediated GSNOR transactivation is dependent on the CArG box. (**A**) Western blot analysis of GSNOR protein level in T/G HA-VSMC cells transfected with Myocardin at the indicated dosages for 24 hours and quantified the western blot data by Quantity One software. β-actin is a loading control. **p < 0.01, *p < 0.05. n = 3. (**B**) The GSNOR promoter (−1264 to +90) containing CArG box element was linked to a luciferase reporter vector (WT-GSNOR-luc). The M-GSNOR-luc represents a luciferase reporter vector including mutated-CArG box GSNOR promoter. (**C**) T/G HA-VSMC cells were transfected with WT-GSNOR-luc or M-GSNOR-luc, together with Myocardin or pcDNA3.1 vector for 24 hours. Then the luciferase reporter assays were used to test the transactivity of GSNOR. The data represent mean ± SEM. **p < 0.01, *p < 0.05. n = 6. (**D**) T/G HA-VSMC cells were transfected with WT-GSNOR-luc, together with si-control orsi-STAT3 in stabled expression Myocardin VSMCs for 24 hours. Then the luciferase reporter assays were used to test the transactivity of GSNOR. The data represent mean ± SEM. *p < 0.05. n = 6. (**E**) T/G HA-VSMC cells were transiently transfected with Myc-Myocardin or a control vector (pcDNA3.1) for 24 hours, and ChIP assays were performed. Sheared DNA/protein complexes were immunoprecipitated by using an anti-Myc antibody (Ab). Then, PCR was carried out to detect the endogenous CArG regions in immunoprecipitated chromatin fragments. The amount of DNA in each sample (input) is shown at the second land. Immunoprecipitations were performed without primary antibody (no Ab) and IgG as a negative. The 2% input and H4 were used as positive control.

Myocardin has been reported to be a key molecular switch that regulates the ability of serum response factor (SRF) to mediate cellular proliferation and differentiation. Myocardin drives transcription by forming a stable complex with SRF binding to CArG box (CC(A/T)_6_GG) in the promoter region^29, 30^. Our data showed that Myocardin overexpression notably enhanced the GSNOR level in T/G HA-VSMC cells by a dose-dependent manner (Fig. 4A). To further determine whether Myocardin-mediated GSNOR transactivation is dependent on the CArG box, we constructed GSNOR gene promoter luciferase reporter vectors containing the deleted or mutant of the CArG boxes. Our data show that deletion or mutation of the CArG boxes in the GSNOR abolished Myocardin-mediated GSNOR promoter activities *in vitro* (Fig. 4B). Knocked out STAT3 in stabled expression Myocardin enhance Myocadin -mediated GSNOR promoter activities in VSMCs (Fig. 4C).

To further confirm the specific binding sites of Myocardin, Chromatin immunoprecipitation (ChIP) assays were performed in T/G HA-VSMC cells which were transfected with Myocardin or Control vector. Cross-linked chromatin was immunoprecipitated with specific antibody (anti-Myc-Myocardin) or no antibody (as negative control). The precipitated chromatin was then purified and amplified by PCR with specific primers of CArG boxes in GSNOR promoters. As shown in Fig. 4D, the negative controls, in which immune-precipitation was performed without antibody (No Ab), showed no any PCR signal. Myocardin can bind to the CArG boxes of the GSNOR promoter. These data reveal that the CArG box identified within the GSNOR promoter binds Myocardin *in vivo*, in T/G HA-VSMC cells. These data demonstrated that Myocardin is a potent nuclear factor which promoted the activities of GSNOR by affecting the formation of the SRF/Myocardin/CArG complex both *in vitro* and *in vivo*.

**Figure 5:**
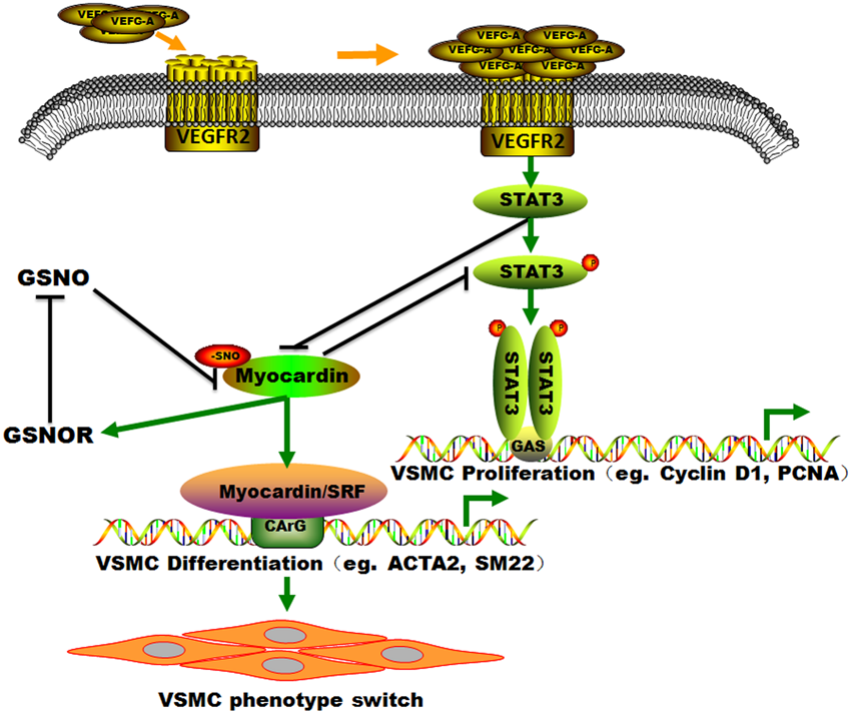
The model that Myocardin, GSNOR and GSNO created a negative feedback loop to regulate the VSMC phenotypic switch. Myocardin positively modulates expression of GSNOR, GSNOR, and then downregulates GSNO and meanwhile GSNO inhibits the contractile markers expression by Myocardin *S*-nitrosylation, thus creating a negative feedback loop to regulate the VSMC phenotypic switch.

In a word, Myocardin positively modulates expression of GSNOR, GSNOR, and then downregulates GSNO and meanwhile GSNO inhibits the contractile markers expression by Myocardin *S*-nitrosylation, thus creating a negative feedback loop to regulate the VSMC phenotypic switch (Fig. 5).

## Discussion

In this study, the major finding is the discovery that VEGF-A activates STAT3 in VSMC phenotypic switch process and that this activation is required for VEGF-A-induced SMC proliferation. In addition, we demonstrated that STAT3 activation is VEGFR2 dependent. Specifically, Myocardin-an interation with STAT3 nuclear transcription factors-can be nitrosylated and inhibited by nitric oxide after VEGFR2 activation induced by VEGF-A. The activity inhibition of Myocardin by VEGF-A is critical for STAT3 expression and the resultant VSMC phenotypic switch. These observations reinforce the importance of nitric oxide and *S*-nitrosylation in VSMC phenotypic switch and angiogenesis. In addition, the findings provide mechanistic insight into the signaling pathways responsible for VEGF-A-induced STAT3 activation in VSMC, strongly suggesting the need for cross-talk between VEGF-A-VEGFR2 and JAK-STAT3 through the “bridge” molecule-Myocardin. Therefore, the Myocardin and STAT3 in VEGF-A-induced VSMC phenotypic switch could provide unique and unexpected therapeutic targets for angiogenesis dependent diseases.

The de-differentiation and proliferation of smooth muscle cells (SMCs) are widely accepted as the major contributor to vascular remodeling^31^. VEGF-A is a particularly important endothelial growth factor associated with tumor progression, wound healing and development. VEGF-A appears to function as a key regulator of physiologic as well as pathologic angiogenesis^32^. VEGF-A can enhance the expression of stem cell markers CD44 and Sox17 in VSMC (Fig. S2). Human VSMC may be stem cell-derived during the culture process. Multipotent vascular stem cell (MVSCs) can differentiate into SMCs and chondrogenic cells, thus contributing to vascular remodeling and neointimal hyperplasia^33^. VEGFR1 has a very important function in VSMC proliferation. VEGFR1 could be upregulated in rat VSMC under hypoxic tress and its activation stimulates VSMC proliferation via STAT3^34, 35^. VEGFR2 is known to mediate the full spectrum of VEGF-A responses in endothelial cells, including cell survival, proliferation, migration and tube formation^9^. Our experimental results show that the effect of VEGF-A on the phosphorylation of VEGFR2 is greater than the phosphorylation of VEGFR1 in VSMCs (data no show). However, until now, no detailed description of the signaling pathways has been published in VEGF-A-VEGFR2-STAT3-dependent VSMC phenotypic switch. Likewise, although VEGF-A-VEGFR2-STAT3 have long been considered critical for VEGF-A-mediated VSMC phenotypic switch and angiogenesis, the exact mechanisms by which Myocardin regulates VEGF-A- dependent VSMC phenotypic switch have until now been unknown. Our results demonstrate that STAT3, but not the STAT1 or STAT5, acts downstream of VEGF-A-VEGFR2 to promote VSMC proliferation.

This discovery increases our understanding of the STAT3 role in VEGF-A-induced VSMC proliferation. However, previous study reported that VEGFA-induced p-VEGFR2 to enhance EC proliferation by exacerbating STAT3 activation, leading to pathological angiogenesis^15^. An activated STAT3 mutant (STAT3C) up-regulates VEGF expression and stimulates tumor angiogenesis, however, the effect could be abrogated when a STAT3-binding site in the VEGF promoter is mutated^21^. Our results also point to the JAK-STAT3 pathway for VSMC proliferation mediated by VEGF-A in VSMC (Fig. 1E). Thus, VEGF-STAT3-VEGF forms a positive feedback loop to regulate smooth muscle phenotype conversion. But the exact mechanism is an avenue of further investigation in VEGF-A-VEGFR2-STAT3-VEGF-A pathway between vascular smooth muscle and endothelial cells via cellular communication, then affect physiological and pathological processes of angiogenesis.

Post-translational *S*-nitrosylation serves as a major mode of protein modification in mammalian cells and a growing body of evidence has shown that transcription factors and their activating pathways are primary modified targets^36^. More than 100 proteins have been reported to be *S*-nitrosylated, including JNK1 and JNK3, resulting in either inhibition or activation of protein function^37^. JNK1 activity is suppressed after nitrosylation on its Cys116 by nitric oxide generated after IFN- administration in macrophages^38^. Conversely, STAT3 *S*-nitrosylation is associated with inducible nitric oxide synthase (iNOS)-produced nitric oxide (NO) and GSNO, exogenous GSNO inhibited STAT3 activation via inhibiting STAT3 phosphorylation in Tyr(705) locus, Cys(259) was the target residue of GSNO-mediated *S*-nitrosylation of STAT3^39^. In our study, Myocardin activity was negatively regulated by *S*-nitrosylation in treating GSNO VSMC (Figs 2B and F and 3A). Myocardin *S*-nitrosylation inhibited the expression of ACTA2, SM22 and Myh11 to regulate the VSMC differentiation (Figs 2G and H and 3D and E). Therefore, Myocardin is sufficient for establishment of a SMC-like contractile phenotype^40^.

Angiogenesis is a complex process that includes recruitment and proliferation of mural cells- SMC and pericytes. Abnormal VSMC activation is associated with various vascular disorders such as atherosclerosis, in-stent restenosis, vein graft disease, and transplantation-associated vasculopathy^41^. Angiogenesis is characterized by the migration and proliferation of vascular endothelial cells and SMC, and the migration and proliferation during angiogenesis is mediated by specific growth factors. Some of the factors are platelet-derived growth factor (PDGF), transforming growth factor-α (TGF-α), fibroblast growth factor (FGF) and VEGF 1 and 2^42^. VEGF-A has been shown to play an important role in angiogenesis and is an endothelial cell chemoattractant. In addition, certain VEGF-A isoforms are implicated in the normal formation of smooth muscle cell-surrounded arteries^43^. VEGF is a secreted mitogen in vascular endothelial cells, and promotes vascular permeability and neovascularization *in vivo* ^10^.

Cell-cell interactions are critical for vascular development. But how the two nuclear transcription factors Myocardin and STAT3 to regulate the intercellular communication need further research via regulating the human vascular endothelial cells and VSMC differentiation and proliferation, migration. Although future study is still needed to determine whether Myocardin and STAT3 contribute coordinately to VEGF-A-mediated neovascularization, this study provides insights into the signaling pathways responsible for VEGF-A dependent angiogenesis and shed more light on the therapeutic programs for cell-based neovascularization therapies against vascular diseases.

## Methods

### Reagents

Recombinant human VEGF-A protein was obtained from R&D Systems and S-Nitrosoglutathione (GSNO) was obtained from santa cruz. Antibodies to pVEGFR2, VEGFR2, pSTAT1, STAT1, pSTAT3, STAT3, pSTAT5 and STAT5 were purchased from Cell Signaling; ACTA2, SM22, Myh11 and Smoothelin antibodies were purchased from Abcam; Myc antibody was purchased from proteintech.

### Plasmids

The promoter region of GSNOR (−1264/+90) were amplified by PCR followed by cloning into pGL3-Basic luciferase reporter vector. The primer used to create GSNOR-luc was as follows: GSNOR: F-5′ CGAGGTACCCAGAAATCCAGTAGGCAGTT 3′ and R-5′ TATACGCGTATGTTCACGGATTCTGGTCG 3′, the vector pGL-3(Pomega) was used as a control.

Additional GSNOR promoter-reporter constructs containing mutations to putative CArG box were generated by site-directed mutagenesis using the QuikChange site-directed mutagenesis kit (Stratagene, La Jolla, CA). The WT-GSNOR-luc CArG box was changed from -CCATAAATAG- to -CACCCATAGG- (M-GSNOR-luc) and these nucleotide mutations abolished myocardin-SRF-binding sites. The primers used were as follows: GSNOR -M-luc: forward 5′: CTGGTAGTCATCTTTCCACCCATAGACGTTAAAAGCCAAA 3′; reverse 5′: TTTGGCTTTTAACGTCTATGGGTGGAAAGATGACTACCAG 3′.

The Myc-Myocardin cDNA constructs were generated by PCR-based cloning with IMAGE cDNA clone from human Myocardin cDNA into pCDNA3.1vector (Addgene), respectively. The mutants of Myocardin constructs were generated by PCR-based mutagenesis for deletion or point mutation.

### Cell Culture and Transient Transfection

T/G HA-VSMC cells (ATCC) were grown in Dulbecco’s modified Eagle’s medium (DMEM) (GIBCO) supplemented with 10% fetal bovine serum at 37 °C in a 5% CO_2_ incubator.

For transient expression experiments with T/G HA-VSMC cells, 60–80% confluent cells were transfected for 3 hours with 2 g of plasmids by using 8 μL of Lipofectamine 2000 and 8 μL of Plus reagent (Invitrogen). One day later, cells were serum starved overnight, and then treated with VEGF-A.

### Immunofluoresence Staining

Cells after treatment were fixed in 4% paraformaldehyde for 20 min, and then blocked with normal goat serum for 20 min at room temperature. Then, rabbit Smooth Mucsle Actin and Myh11 (Abcam) antibodies were added and incubated in a humid chamber overnight. After washing with PBS twice, cells were incubated with appropriate secondary antibodies (fluorescein isothiocyanate (FITC)-goat anti-rabbit, FITC-goat anti-mouse) for 30 min at 37 °C. After washing with PBS, the samples were observed by laser scanning confocal microscopy (Olympus). 4′,6-diamidino-2-phenylindole (DAPI) stain (blue) highlights the total nuclei.

### Luciferase Reporter Assays

Luciferase assays were performed as described previously^44^. After transfection for 24 hours, luciferase activity was measured by a Synergy 4 (Bioteck). Transfection affeciencies were normalized to total protein concentration of each luciferase assay preparation. All experiments were performed at least three times with different preparations of plasmids and primary cells, producing qualitatively similar results. Data in each experiment are presented as the mean ± standard deviation of triplicates from a representative experiment.

### siRNA Design and Transient Transfection

siRNAs for VEGFR2, STAT1, STAT3, and STAT5 were designed with BLOCK-iT RNAi designer (www.invitrogen.com). Sequences are available in SI Materials and Methods. The transfection of siRNA into T/G HA-VSMC cells was performed with Lipofectamine 2000 following the manufacturer’s protocol for T/G HA-VSMC cells.

### Immunoprecipitation and Western Blotting Analysis

Cells were harvested in lysis buffer and equal amounts of proteins were incubated with a specific antibody overnight at 4 °C with gentle rotation. Protein A/G Plus-agarose beads (Santa Cruz Biotechnology) were used to pull down the antibody complexes. Immune complexes were then separated by SDS/PAGE and analyzed by Western blotting.

### Biotin Switch Assay

Experiments were performed following the methods previously described in ref. 45.

### *In Vitro* Phosphatase Assay

*In vitro* phosphatase reactions were carried out on Myc-Myocardin protein immunoprecipitated from transfected cells, and results were visualized by Western blot analysis with phospho specific STAT3 antibodies.

### Statistical Analysis

Data are shown as mean ± SD for 3 or 4 separate experiments. Differences were analyzed by Student’s t test. Values of *P* < 0.05 were considered statistically significant.

## Electronic supplementary material

Supporting Information
