# Supplementary material for: VEGF-A Stimulates STAT3 Activity via Nitrosylation of Myocardin to Regulate the Expression of Vascular Smooth Muscle Cell Differentiation Markers
Source: Sci Rep. 2017 Jun 1;7:2660. doi: 10.1038/s41598-017-02907-6 (PMC5453982; doi:10.1038/s41598-017-02907-6)
Supplement: Supplementary file 1 — Supporting Information [file 41598_2017_2907_MOESM1_ESM.doc]

**VEGF-A Stimulates STAT3 Activity via Nitrosylation of Myocardin to Regulate the Expression of Vascular Smooth Muscle Cell Differentiation Markers**

Xing Hua Liao^1, 2a*^, Yuan Xiang^1a^, Hui Li^1a^, De Liang Zheng^2^, Yao Xu^1^, Cheng Xi Yu^1^, Jia Peng Li^1^, Xiao Yu Zhang^1^,Wei Bin Xing^1^, Dong Sun Cao^1^, Le Yuan Bao^1^, Tong Cun Zhang^1, 2*^

**Supporting Information**
**SI Materials and Methods**
**siRNA Design and Transient Transfection.**

siRNAs for bovineVEGFR2, STAT1, STAT3, and STAT5 were designed with BLOCK-iT RNAi designer (http://rnaidesigner.thermofisher.com/rnaiexpress/). VEGFR2 siRNA1 sequence is CCAUGUCUCGGGUCCAUUU, VEGFR2 siRNA2 sequence is GCUUUACUAUUCCCAGCUA, VEGFR2 siRNA3 sequence is GGGAAUACCCUUCUUCGAA. STAT1 siRNA sequence is GCAGGUUCACCAGCUUUAU. STAT5 siRNA sequence is GGUGAAGACUCUGUCCCUA. STAT3 siRNA sequence is a mixture of (i) CCACUUUGGUGUUUCAUAA; (ii) GCAACAGAUUGCCUGCAUU; (iii) GCCCAACAUCUGCCUAGAU. The control siRNA sequence is CGGGAACUACAAGACACGUGCUGAA.

The transfection of siRNA into T/G HA-VSMC cells was performed with Lipofectamine 2000 following the manufacturer’s protocol for T/G HA-VSMC cells. After cells were confluent for 1 days, T/G HA-VSMC cells were trypsinized and spun down. A total of 1×10^6^ cells were resuspended in 100ｕL of room-temperature DMEM and mixed with 100 pmol of siRNA. The cell/DNA suspension was then transfected with transfection reagent (Lipofectamine 2000) according to manufacturer’s instructions. After incubation for 6 hours, the medium was removed and replaced with normal culture medium for 24 hours. The efficiency of transfection was evaluated with pmaxGFP expression.


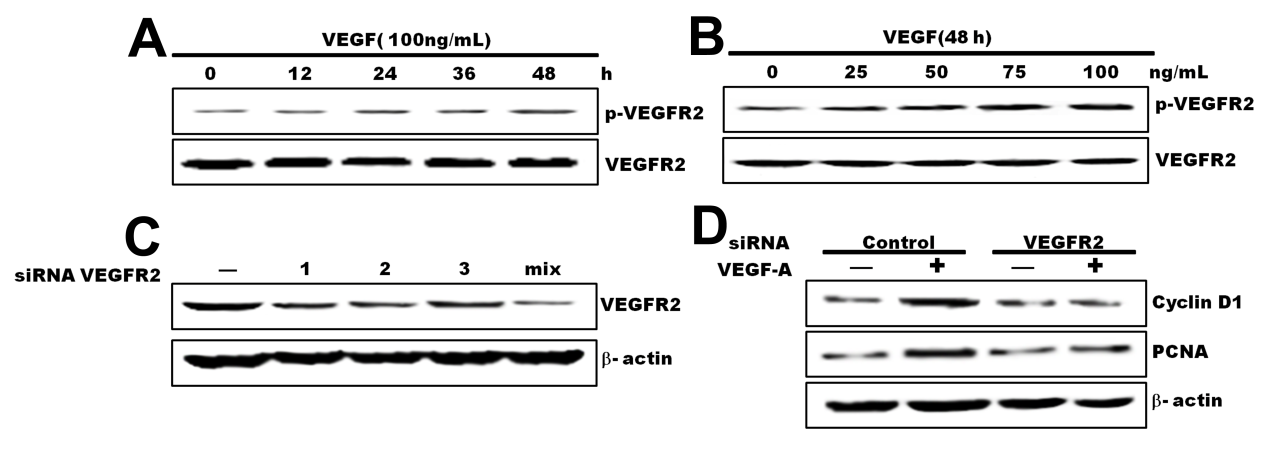


**Fig. S1. VEGFR2 is activated by VEGF-A.**

(A) Western blot analysis of VEGFR2 phosphorylation in T/G HA-VSMC cells treated with 100 ng/mL VEGF-A for indicated time periods. (B) Western blot analysis of T/G HA-VSMC cells treated with VEGF-A with the indicated dosages for 48 hours. (C) Western blot analysis of VEGFR2 protein level in T/G HA-VSMC cells transfected with VEGFR2 siRNA1, -2, -3, or a mixture of the three. (D) Western blot analysis of Cyclin D1 and PCNA protein level in T/G HA-VSMC cells transfected with VEGFR2 siRNA.

**
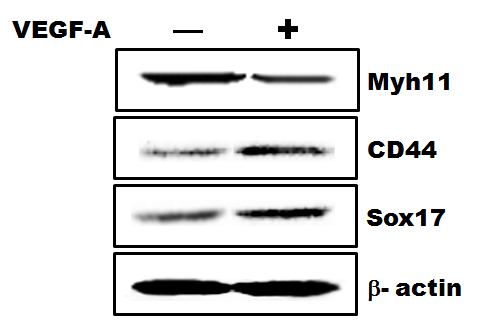
**

**Fig. S2. VEGF-A enhance the expression of stem cell markers CD44 and Sox17.**

Western blot analysis of CD44 and Sox17 in T/G HA-VSMC cells treated with 100 ng/mL VEGF-A for 48 hours.
